# Effect of two esthetic digitally produced materials used in fabrication of extracoronal attachments on the stresses Induced in removable partial dentures

**DOI:** 10.1186/s12903-024-04477-2

**Published:** 2024-07-04

**Authors:** Dina Abd El Moez Abd Allah, Noha Helmy Nawar, Ahmed Mostafa Abdelfattah

**Affiliations:** Oral and Maxillofacial prosthodontics Department, Faculty of Dentistry, Ain Shams University, African unity organization street, Cairo, 11561 Egypt

**Keywords:** Stress analysis, Polyetheretherketone, Computer aided design, Computer assisted manufacturing, Zirconium oxide

## Abstract

**Background:**

Preservation of the remaining structures while maintaining an esthetic appearance is a major objective in removable partial prosthodontics. So, the aim of the current study was to compare the stresses induced on the supporting structures by two digitally produced esthetic core materials; Zirconia and Polyetheretherketone when used as an extracoronal attachment in distal extension removable partial dentures using strain gauge analysis.

**Methods:**

A mandibular Kennedy class II stone cast with the necessary abutments’ preparations was scanned. The mandibular left canine and first premolar teeth were virtually removed. An acrylic mandibular left canine and first premolar teeth were prepared with heavy chamfer finish line and scanned. Virtual superimposition of the acrylic teeth in their corresponding positions was done. Two strain gauge slots were designed: distal to the terminal abutment and in the residual ridge. Two models and two sets of scanned teeth were digitally printed. The printed teeth were then placed in their corresponding sockets in each model and scanned. The attachment design was selected from the software library and milled out of Zirconia in the model ZR and Polyetheretherketone in the model PE. Five removable partial dentures were constructed for each model. The strain gauges were installed in their grooves. A Universal testing machine was used for unilateral load application of 100 N (N). For each removable partial denture, five measurements were made. The data followed normal distribution and were statistically analyzed by using unpaired t test. P value < 0.05 was considered to be statistically significant.

**Results:**

During unilateral loading unpaired t test showed statistically significant difference (*p* = 0.0001) in the microstrain values recorded distal to the abutment between the models ZR (-1001.6 µε ± 24.56) and PE (-682.6 µε ± 22.18). However, non statistically significant difference (*p* = 0.3122) was observed in the residual ridge between them; ZR (16.2 µε ± 4.53) and PE (15 µε ± 3.74).

**Conclusions:**

In removable partial dentures, Polyetheretherketone extracoronal attachment induces less stress on the supporting abutments compared to the zirconia one with no difference in the stresses induced by them on the residual ridge.

## Background

The increase in patients’ concerns about their appearance in the society has increased their desire for esthetic dental restorations. Such a desire not only challenges the dentists to provide functional care but they have to provide esthetic care as well [1]. Among such challenges is the non-esthetic display of the metal clasps in the conventional clasp retained removable partial denture whenever the patient smiles or speaks. To overcome such challenges, different approaches have been considered as providing lingual retention and veneering the metal clasp arm with acrylic or composite resins. However, frequent debonding and the high failure rate ended up in their limited applications [2]. Implant supported prosthesis may also offer a suitable esthetic solution yet, the placement of dental implants is not always a feasible option because of the anatomical, financial and psychological limitations [3, 4]. However, extracoronal attachments provide support and retention for removable partial dentures (RPDs) meanwhile providing esthetics as well [5–7]. They also provide a biomechanical advantage in long-span removable partial dentures [6, 7]. On the other hand, they are expensive and require extensive abutment teeth preparation compared to the conventional clasp retained RPD. Meticulous and complex clinical and laboratory procedures are needed too. Moreover, attachment placement is dependent on several factors as the available clinical crown height of the abutment teeth, available interarch space, number of supporting abutments and their periodontal condition [8–10].

One of the most commonly used materials for attachment fabrication is the Cobalt chromium (Co-Cr) alloy. Such an alloy has revealed good clinical results due to its high modulus of elasticity, hardness and low cost [9]. Thanks to the wide applications of the digital technology in the dental field, esthetic materials as Zirconia and Polyetheretherketone (PEEK) can be used nowadays for fabrication of different prosthesis with high precision and passive seating [10–12]. Zirconia was used for fabrication of bridges, double crowns and extracoronal attachments due to its biocompatibility, good esthetics, high hardness, durability and wear resistance [9, 13–15]. High-strength polymeric resins as the PEEK material have been introduced as a promising alternative to ceramic materials [10]. Such a material has been described to have unique resilient properties, shock absorption, biocompatibility, corrosion resistance, minimal creep and modulus of elasticity similar to bone [10, 11, 16, 17]. Several studies investigated the PEEK material when used as implant supported frameworks, posts in endodontically treated teeth as well as clasps in RPD and gave promising results [18–22].

Stress analysis studies were used for evaluation of the stress patterns induced by Co-Cr, Zirconia and PEEK materials in RPD. Sadek et al. concluded that PEEK could be a material of choice for restoring free end partially edentulous cases due to its superior biological and mechanical properties [12]. Furthermore, Saleh et al. used strain gauge analysis to compare the stresses induced by different Co-Cr attachment designs [7]. Jagodin et al. also showed that zirconia can be used as an extracoronal attachment in RPD provided that a suitable attachment design is selected [14]. Moreover, Orujov et al. concluded that the tooth preparation and the mesiodistal length of the extracoronal attachment made of zirconia affect the stresses developed at the tooth attachment interface [16].

However, the stresses induced by PEEK when used as an extracoronal attachment material in distal extension removable partial denture were not assessed and mentioned in the literature. So, the current study was conducted to compare the stresses induced on the supporting abutments and the residual ridge by two esthetic core materials; zirconia and polyetheretherketone when used as an extracoronal attachment in distal extension removable partial dentures using strain gauge analysis. The null hypothesis was that no difference existed in the stresses induced on the supporting abutments and the residual ridge by both materials in attachment retained distal extension RPD.

## Methods

**Fig. 1:**
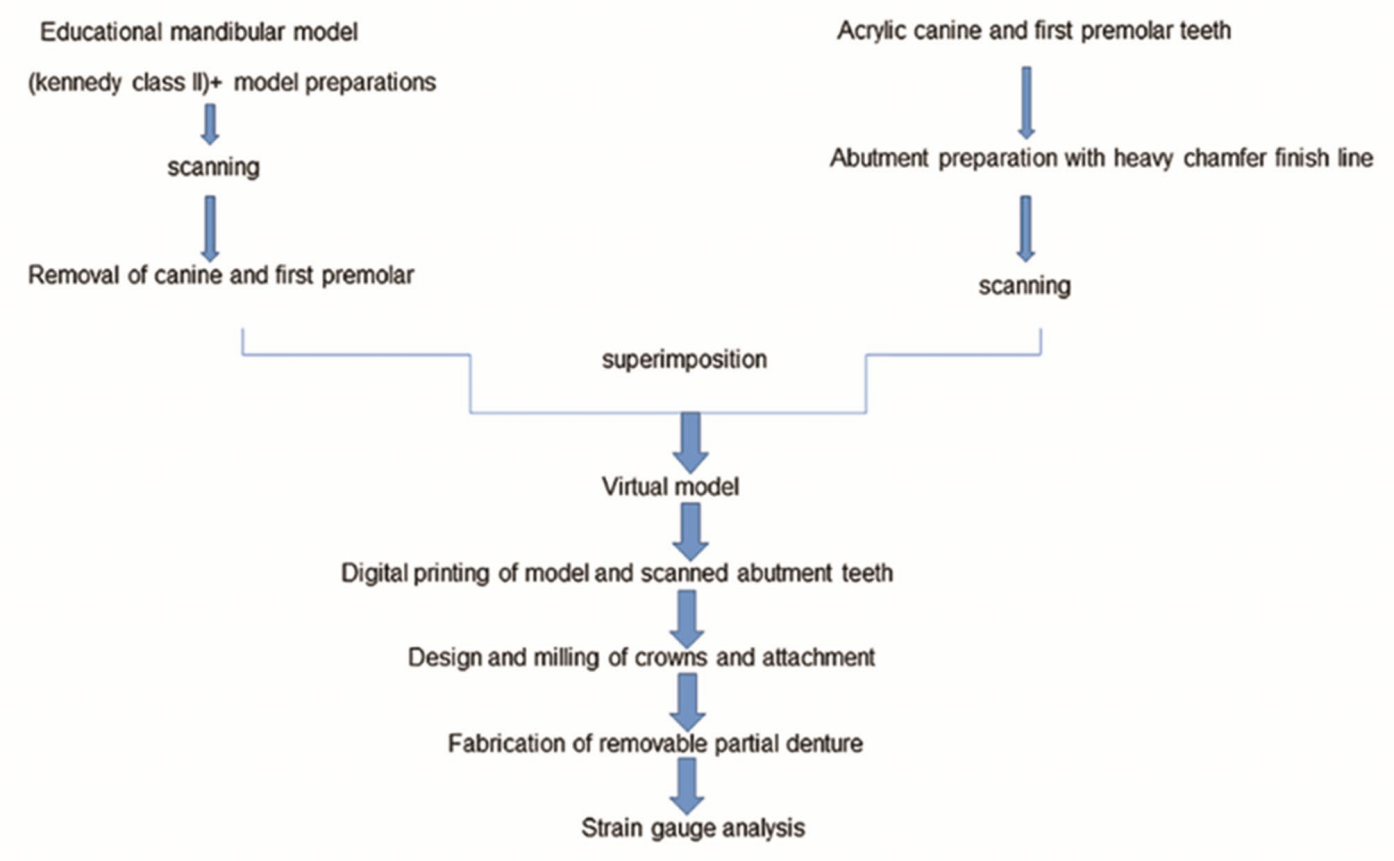
Flowchart showing the study workflow

The current study was conducted using a digitally produced mandibular Kennedy class II model [23]. The terminal abutments in the free end saddle side were the mandibular left canine and the first premolar teeth. Two models were used in which the attachment was made out of zirconia in the model ZR while in the model PE, it was made out of PEEK. Five removable partial dentures were made in each group (Fig. 1). The sample size was calculated in the light of the results published by Emera et al. It was based on 95% confidence interval and power 80% with α error 5% (MedCalc® version 12.3.0.0 program, Ostend, Belgium) [24].

**Fig. 2:**
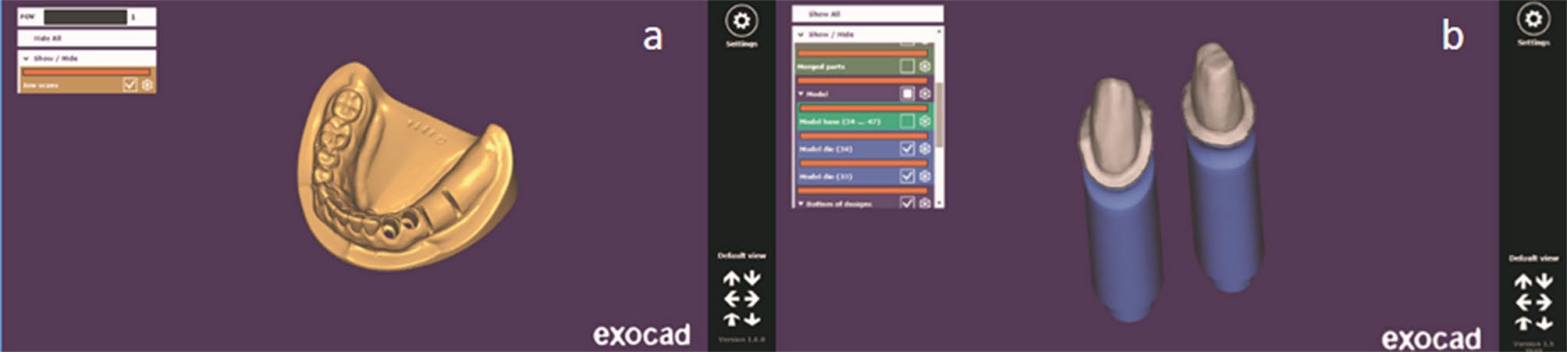
a. Diagram showing the virtually designed model having the sockets of abutment teeth and the strain gauge slots. b. Diagram showing the virtually designed abutment dies having a heavy chamfer finish line

For model construction, an educational mandibular Kennedy class II stone cast (Ramses medical products, Cairo, Egypt) was used. Preparations for rest seats were made in the right mandibular second premolar and the mandibular first molar to receive a Double Aker clasp [25]. The cast was then scanned (DOF swing scanner, DOFlabs, Seoul, South Korea) and a standard tessellation language (STL) file of the virtual cast was produced. Using the CAD software (Exocad Dental CAD, Exocad Inc. Darmstadt, Germany), the mandibular left canine and first premolar teeth were virtually removed from the virtual cast by Boolean subtraction. Meanwhile a mandibular left canine and first premolar acrylic teeth (Ramses medical products, Cairo, Egypt) were prepared to have a heavy chamfer finish line [10]. A Secondary plane of reduction was made on the labial and buccal surfaces of the teeth. The prepared mandibular teeth were scanned and the STL file was produced [10]. Using the CAD software, the scanned acrylic teeth were virtually superimposed in their corresponding sockets. A space of 0.25 mm was left between the inner surface of the socket and each tooth root surface simulating the periodontal membrane space. A 2 mm layer thickness was removed from the residual ridge crest of the scanned model representing the mucosal layer that was added later [26, 27]. Two strain gauge slots were designed on the software to receive the strain gauge rosettes. The first one was placed distal to the socket of the terminal abutment and the second slot was placed posteriorly in the edentulous ridge (Fig. 2a) [26, 27]. The design of the virtual model was checked and the STL file was sent to the 3D printing machine(Form2 3D printer, formlabs, Somerville, Massachusetts, United States). Similarly, two pairs of the prepared mandibular left canine and mandibular left first premolar were digitally printed (Fig. 2b). The printed model and prepared teeth were then checked for any defects and the teeth were placed in their sockets.

**Fig. 3:**
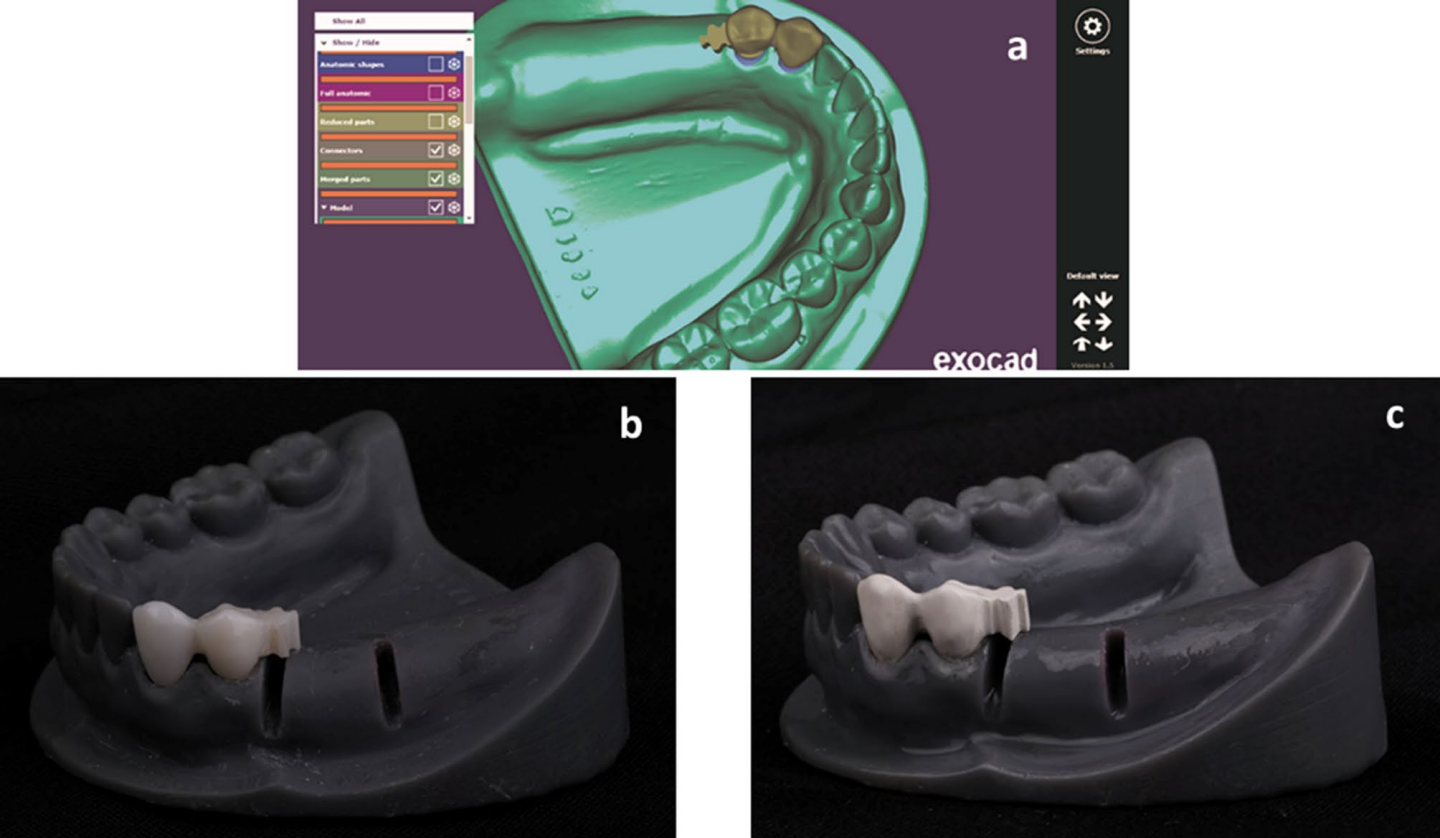
a. Top view for the virtual placement of the selected attachment design showing lingual preparation for the lingual guiding arm of the removable partial denture on the lingual side of the mandibular left first premolar. b. Lateral view for the 3D printed model with the milled zirconia attachment. c. Lateral view for the 3D printed model with the milled PEEK attachment

The whole model was then scanned and two splinted crowns were designed (Exocad Dental CAD, Exocad Inc. Darmstadt, Germany) for the prepared abutments. The lingual surface of the mandibular left first premolar crown was designed to have a shoulder finish line at the junction between the middle and gingival parts [4].The attachment (vario soft 3 mini sv, Bredent medical group Gmbh, Germany) was chosen from the software library and attached to the distal wall of the mandibular left first premolar crown [14, 28, 29]. The attachment was placed on a line bisecting the angle between the crest of the ridge and the sagittal plane of the model (Fig. 3a) [30]. In the model ZR, the crowns and the attachment were milled out of zirconia (katana, Kuraray Noritake Dental, Inc, Okayama, Japan) in a five axis milling machine(DWX-52D, Roland DGA, California, USA) and sintered(sintering temperature 1650 ^o^c, total process time 239 min) (Fig. 3b). However, in the model PE they were milled out of PEEK (Brecam Biohpp, Bredent medical group Gmbh, Germany) (Fig. 3c). The crowns and the attachments were then checked for perfect fit with the prepared abutments and cemented in place with temporary cement (Cavex temporary cement, Cavex Holland BV, Netherlands).

After attachment cementation, the edentulous ridge in the model was covered by two layers of modeling wax (Cavex Holland BV, Netherlands) to compensate for the 2 mm height subtracted from the crest of the residual ridge during virtual model designing. Each model was then duplicated and an acrylic sheet (Bio-Art Equipamentos Odontologicos Ltda, Brasil) was then pressed on the duplicate cast using a vacuum press machine (Yates Motloid, United States of America). Light body silicone rubber base impression material (Speedex, C-silicone, Coltene, Coltene whaledent Inc., Altstätten, Switzerland) was placed on the crest of the residual ridge of the model and the acrylic template was then seated [26]. The mucosa simulating layer was then bonded in place using a cyanoacrylate adhesive (Amir Alpha Co., Cairo, Egypt).

**Fig. 4:**
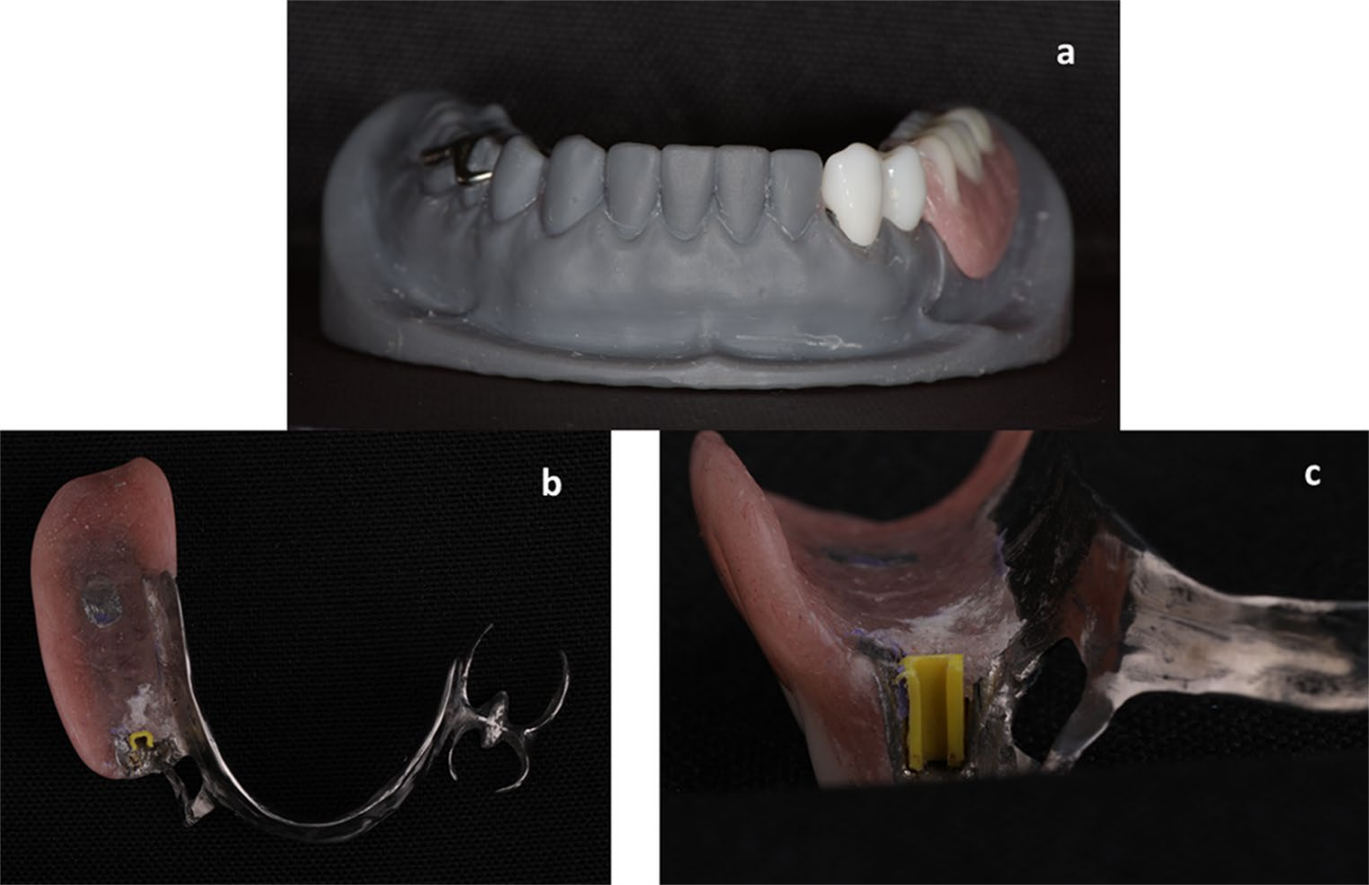
a. (Frontal view) of the 3D Printed model with zirconia attachment and the removable partial denture. b. Fitting surface of the removable partial denture showing the matrix part of the attachment. c. The matrix part of the attachment; 4.1 mm depth, 2.6 mm width, 6.0 mm length, yellow color indicating regular retention

Each model was then duplicated into a refractory cast for RPD fabrication. For the RPD design, a combined denture base was used in addition to a Double Aker clasp placed on the mandibular right second premolar and first molar teeth. The lingual bar was used as a major connector. Lost wax technique was used for RPD fabrication(Fig. 4a). The female part (Bredent medical group Gmbh, Germany) was then picked up in the RPD with cold cure acrylic resin material (Duralay, Interfloor, Haslingden, Lancashire, UK) (Fig. 4b) (Fig. 4c).

**Fig. 5:**
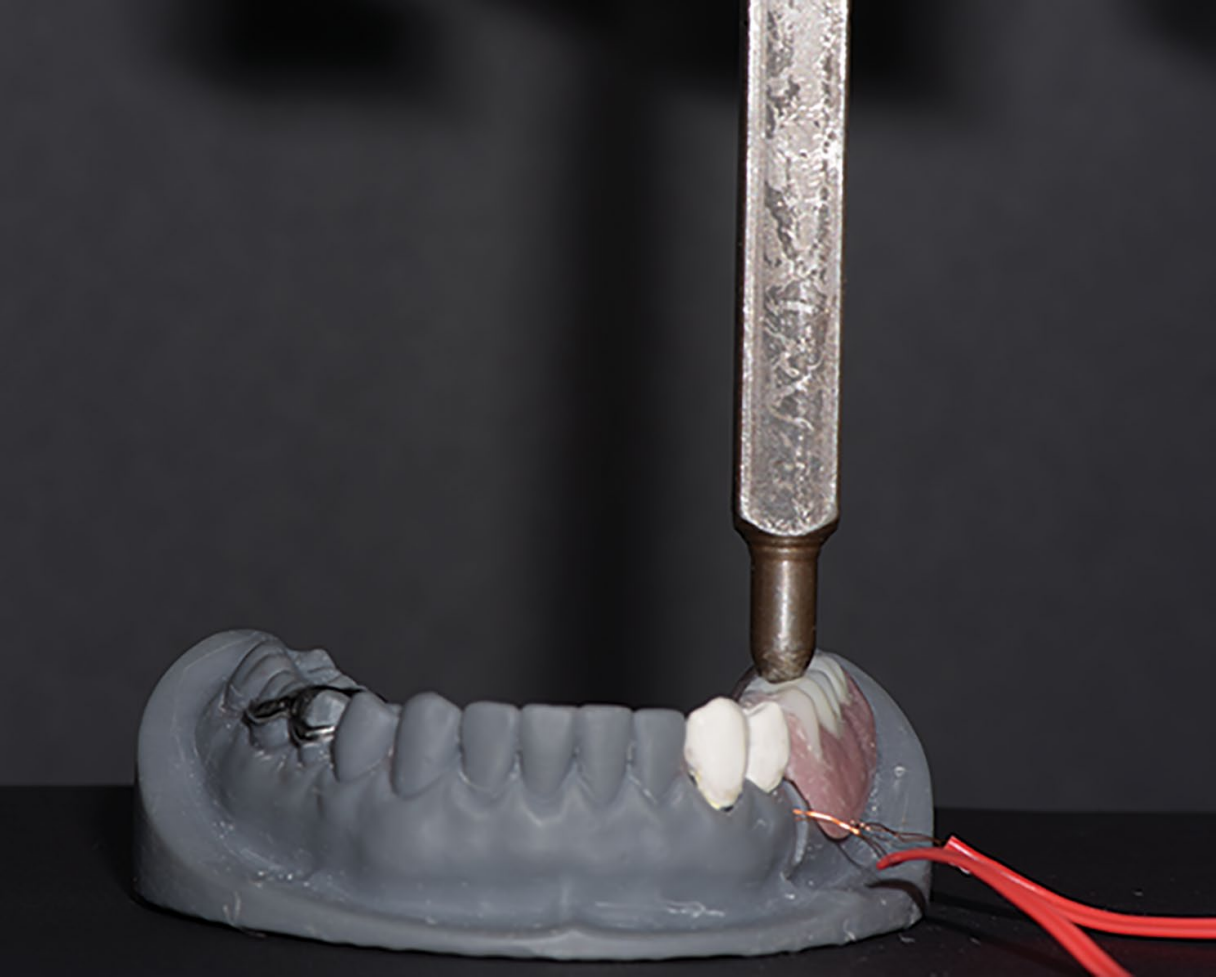
Unilateral load application of 100 N on the central fossa of the mandibular left first molar using the Universal testing machine

The strain gauges rosettes (KFG-1-120-C1-11L1M2R; Kyowa Electronic Instruments Co., Ltd., Tokyo, Japan; resistance 119.6 ± 0.4% Ω; length:1 mm; factor: 2.08 ± 1.0%) were placed in their grooves on the distal aspect of the abutment and the edentulous ridge perpendicular to the occlusal plane [26, 27]. A delicate layer of cyanoacrylate adhesive (Amir Alpha, Cairo, Egypt) was used to bond the rosettes in place. The terminals of the strain gauge wires were attached to a sensor interface board of a quarter bridge circuit four channel strain meter (Kyowa sensor interface PCD-300 A; Kyowa Electronic Instruments Co., Ltd, Tokyo, Japan) to convert the electro impulses to micro strain [31]. Each model was placed on the lower plate of the universal testing machine (Lloyd LRX; Lloyd Instruments Ltd., Fareham, UK) with cell load 5000 N. For calibration 10–60 N loads were applied five times in 10 N steps [26]. For unilateral loading, an I bar shaped load applicator was placed on the central fossa of the mandibular left first molar tooth. The central fossa was notched in each overdenture to replicate the position of load application (Fig. 5) [26]. The magnitude of the applied load was 100 N and was increased from 0 to 100 N at a constant rate of 0.5 mm/min [26, 31]. Once the load was applied, the software (PCD-30 A; Kyowa Electronic Instruments Co., Ltd, Tokyo, Japan) was converted the microvoltage output into a microstrain reading using the formula $e\theta \, = \,1/4 \cdot (\Delta R)/R.E\, \equiv 1/4\,.Ks.\varepsilon .E$

**(**e; output voltage, R; resistance, ΔR; resistance changes due to strain, E; excitation voltage, K: gauge factor). For each removable partial denture, five measurements were made. Five minutes recovery period was permitted between the measurements [26].

For blinding, the models were coded by a second party rather than the authors before the measurements were made. The measurements were made by a single operator in the Biomaterials laboratory available in author’s university who was instructed about the site and magnitude of load application. A trial measurement for demonstration to the laboratory operator was made before the actual measurements were made. Once the load was completely applied, the data were analysed using the software (PCD-300 A Kyowa Electronic Instruments Co., Ltd, Tokyo, Japan) and the microstrain values were recorded. The secret codes were then decoded and the results were given to the authors.

Statistical analysis was performed using the statistical package for social sciences, version 21.0 (SPSS Inc., Chicago, Illinois, USA). The recorded data followed normal distribution as indicated by Kolmogrov-Smirnov normality test. Paired and Un-paired T tests were used for intra and inter group comparisons respectively. The significance level was set at *P* < 0.05. Negative values indicated compressive strain while positive values indicated tensile strain.

## Results

During unilateral loading, compressive and tensile strains developed distal to the abutment and in the residual ridge respectively in both models. Moreover, unpaired t test revealed a statistically significant difference in the microstrain values developed distal to the abutment in both models (P-value = 0.0001, T-value = 47.21). However, there was no statistically significant difference in the microstrain values developed in the residual ridge between them (P-value = 0.3122, T-value = 1.02). Meanwhile, paired t test showed a statistically significant difference in the microstrain values developed distal to the abutment and the residual ridge in each model. The microstrain values are listed in Table (1).

**Table 1:** Microstrain values recorded distal to the abutment and the residual ridge in both models during unilateral loading

	Location of strain gauge rosette	Distal to the abutment	Residual ridge		
		X(µε) SD	X(µε) SD	T value	*P* value
Model ZR		-1001.6 ± 24.56 ^Aa^	16.2 ± 4.53 ^Ab^	200.1	0.0001
Model PE		-682.6 ± 22.18 ^Ba^	15 ±3.74 ^Ab^	145.9	0.0001
T value		47.21	1.02		
*P* value		0.0001	0.3122		

Different superscript lowercase letters in rows indicate significant difference regarding the microstrain values recorded distal to the abutment and the residual ridge in the same model while different superscript uppercase letters in columns indicate significant difference between the models regarding the microstrain values recorded distal to the abutment and the residual ridge.

X, mean; SD, standard deviation; µε, microstrain unit. Negative sign indicates compressive strain, positive sign indicates tensile strain.

## Discussion

Esthetic core materials as Zirconia and PEEK have been used for the fabrication of dental attachments in prosthodontics [12, 14, 15]. However, the stresses induced by them when used as an extracoronal attachment in distal extension removable partial denture were not compared and mentioned in the literature. So, the current study was conducted to compare the stresses induced on the supporting abutments and the residual ridge by such materials when used as an extracoronal attachment in distal extension removable partial dentures using strain gauge analysis. The null hypothesis was partially rejected as there was a statistically significant difference in the stresses induced in the supporting abutments between both materials. Meanwhile, there was no statistically significant difference in the stresses induced in the residual ridge. The microstrain values recorded distal to the abutment for the model PE were lower than the model ZR. This could be attributed to the shock absorbing property of the PEEK material [10, 11, 17, 18]. Such a result came in accordance with the results of Keiling et al. in which PEEK delivered less stresses to the underlying abutments in partial dentures [32]. Similarly, Emera et al. concluded that PEEK telescopic overdenture abutments induced less peri-abutment strain compared to Zirconia [24]. Tzu-Yu Penget al, also reported less stresses being delivered to the underlying abutments when PEEK was used as a clasp material in RPD compared to other materials [21]. Moreover, Sirandoni et al., showed less stresses at the site of load application in implant supported PEEK frameworks compared to more rigid frameworks as Titanium [22]. Its elastic modulus being closer to bone and acrylic resin compared to zirconia may have also helped with better stress distribution [16, 20]. The less stresses reported in the previously mentioned studies were also related to the shock absorbing property of PEEK in addition to the good stress distribution [19–23, 25, 35].

However, regarding the stresses induced in the residual ridge, there was no statistically significant difference between both models. This can be explained in the light of the results published by Sirandoni et al. [22] and Lee et al. [33] who reported that the shock absorbing property of the PEEK material is limited to the site of load application and where the stress is compressive in nature. Accordingly higher stresses were recorded in the regions distant from the PEEK attachment and where the direction of the developed stresses changed from being compressive distal to the abutment to being tensile in the residual ridge. Furthermore the insignificant difference between both models in the stresses developed in the residual ridge could be attributed to the low elastic modulus of the PEEK material that may have generated a larger bending moment of the removable partial denture and more denture movement under functioning loads and consequently higher bending forces on the residual ridge [33, 34]. Moreover, PEKK material was stated not to allow even distribution of the functional load leading to areas that bear high loads and others that bear lesser loads [26, 27].

Distal to the abutment, the application of unilateral loading in the current study resulted in compressive and tensile micro strains in the supporting abutments and the residual ridge respectively in both models. Such a result matches the results published by saleh et al. [7], chen et al. [34] and Jin Suk Yoo et al. [35]. This could be explained in the light of the difference in the compressibility between the resilient mucosa and partial denture abutment that caused rotational movement of the partial denture during load application [27, 33–35]. Paired T test showed a statistically significant difference in the microstrain values developed in each model for both groups; being higher distal to the abutment compared to the residual ridge. Such a finding comes in line with further studies that reported higher stress levels in the abutments supporting the extracoronal attachments compared to the residual ridge in attachment retained distal extension RPD [36, 37].

In the current study, the models were virtually designed and printed to allow standardization between the models in both groups regarding the exact position of the abutment teeth and placement of strain gauge slots in relation to them. Furthermore, the slots were made uniform and smooth helping to avoid strains that may result from rough surfaces [23, 38]. The design of the attachment was selected in the light of the manufacturer recommendation for zirconia and PEEK materials as its shear distribution property provides protection for the supporting structures and to avoid further designs that may affect the strain values in the current study [14, 28]. The lingual guiding arm was used in the RPD design as it shares some of the loads transmitted to the supporting structures [4]. The lingual bar major connector provided the cross arch stabilization needed to reduce the buccolingual rotation of the RPD and to control the stresses delivered to the abutments [38]. Temporary cement was used in the current study while, in the clinical setting a permanent cement is usually used; a factor that may affect stresses delivered to the abutments compared to the temporary cement. However, the temporary cement in the current study was not a variable.

Clinically, preservation of the remaining oral structures, minimizing the stresses delivered to the abutments and the residual ridge in addition to providing a satisfactory esthetic result are the major objectives in rehabilitating partially edentulous patients with distal extension saddles. Extracoronal attachments provide an esthetic solution for such rehabilitation. Meanwhile, in the light of the current study results, the PEEK material compared to the Zirconia material could be clinically speculated to cause less stress and minimize the torque delivered to the supporting abutments at the same time of providing satisfactory esthetic quality when being used as an extracoronal attachment in the distal extension removable partial dentures. Accordingly, the supporting abutments could stand and last for a long period of time. So, a durable and esthetic prosthetic rehabilitation could be expected; a condition that may lead to a better patient satisfaction and improved quality of life. Although Gagodin et al. [14] compared failure rates and modes for different designs of zirconia attachments compared to the metallic ones under simulated dynamic loading, yet data are limited for PEEK extracoronal attachments. Accordingly, further studies in that scope are needed.

Although the authors managed to standardize fabrication of the models in the current study, site and magnitude of load application in addition to the position of strain gauge rosettes yet the current study offers a number of limitations. First of all, the effect of static axial loading was investigated. However, the human masticatory loads are dynamic and complex in nature. Furthermore, there is variation in the magnitude of load application in the studies related to the rehabilitation with removable partial dentures in the literature. Dahab et al., used 200 N in their study to analyze the stresses on the mandibular unilateral distal extension saddle [39]. Meanwhile, Bhattacharya, D et al., used 100–125 N in their study applied on bilateral distal extension implant-assisted removable partial dentures [40]. However, the 100 N load was the one selected in the current study in the light of Fahmy MM et al. study, applied on the removable partial dentures with 100 N load [27]. Moreover, acrylic resin; the material used for model fabrication, is limited in simulating the mechano-biological nature of the human bone. Furthermore, Zirconia and PEEK are esthetic core materials that need further lamination; a procedure that was not performed in the current study. So, further preclinical investigations evaluating the bond strength of Zirconia and PEEK with the overlying laminate materials under simulated aging conditions should be done. Randomized clinical trials comparing the clinical performance of extracoronal attachments made of Zirconia and PEEK in free end RPDS need to be designed and executed as well.

## Conclusions

In removable partial dentures, Polyetheretherketone extracoronal attachment induces less stress on the supporting abutments compared to the zirconia one with no difference in the stresses induced by them on the residual ridge.

## Data Availability

The dataset used for calculation of the stress value is available from yhe corresponding author upon request.
